# A 3D‐Bioprinted, Cell‐Guiding Hydrogel Patch Promotes Functional Repair of Abdominal Incisions via Anisotropic ECM Remodeling

**DOI:** 10.1002/smmd.70056

**Published:** 2026-09-23

**Authors:** Tianxing Gong, Hao Li, Xueqiang Peng, Xinwei Liu, Xinyan Zhang, Qiushi Tang, Stephanie Willerth, Mario Taba Junior, Ricardo M. Carvalho, Hailong Yu, Shibo Wei, Hangyu Li

**Affiliations:** ^1^ Department of Biomedical Engineering Shenyang University of Technology Shenyang China; ^2^ Department of General Surgery The Fourth Affiliated Hospital of China Medical University Shenyang China; ^3^ Department of Orthopedics General Hospital of Northern Theater Command Shenyang China; ^4^ Chinese Journal of Practical Surgery China Medical University Shenyang China; ^5^ Department of Mechanical Engineering University of Victoria Victoria British Columbia Canada; ^6^ Department of Oral & Maxillofacial Surgery University of São Paulo São Paulo Brazil; ^7^ Department of Oral Biological and Medical Sciences University of British Columbia Vancouver British Columbia Canada; ^8^ Shenyang Key Laboratory of Biomedicine and Intelligent Patch China Medical University Shenyang China; ^9^ Department of General Surgery The First Affiliated Hospital of Jinzhou Medical University Jinzhou China; ^10^ Liaoning Province‐Zimbabwe Belt and Road Joint Laboratory on Biomedical Data Sharing and Cancer Prevention and Control Jinzhou Liaoning China

**Keywords:** 3D bioprinting, abdominal wall repair, anisotropic design, guided tissue regeneration, incisional hernia

## Abstract

Incisional hernias result from disorganized, mechanically weak scar tissue following abdominal surgery. Current surgical meshes are suboptimal and fail to guide functional regeneration, creating a critical need for new therapeutic approaches. This study introduces an architecture‐guided regenerative repair strategy that enhances abdominal incision repair. We developed a Hydrogel‐based Architecture‐guided Regenerative Patch (HARP) featuring 3D‐bioprinted, parallel‐aligned filaments that are oriented perpendicular to the incision to provide architectural guidance. The HARP was fabricated from a novel gelatin/dialdehyde cellulose bioink loaded with fibroblasts and TGF‐β1 and evaluated in vitro and in a rat abdominal incision model. In vivo, the HARP accelerated healing and yielded a twofold increase in mechanical strength over controls. This resulted from the guided deposition of a dense, anisotropic, type I collagen‐rich matrix that resembled native tissue. Our findings support a role for architectural guidance in functional abdominal wall repair, providing a proof of concept for a regenerative repair strategy with the potential to reduce incisional hernia risk.

## Key Points

- A bioactive Hydrogel‐based Architecture‐guided Regenerative Patch (HARP) was 3D‐bioprinted using a gelatin/dialdehyde cellulose ink loaded with fibroblasts and TGF‐β1.
- The patch's anisotropic architecture guides dense, native‐like collagen deposition, yielding a twofold increase in mechanical strength in vivo.
- This study supports an architecture‐guided regenerative repair strategy that enhances abdominal incision repair.

## Graphical abstract

Abdominal incisions can heal into mechanically weak, disorganized scars. We developed a 3D‐bioprinted hydrogel patch with cell‐laden filaments oriented perpendicular to the incision, providing a guiding scaffold for local repair cells. This architecture supports organized collagen deposition and improves mechanical restoration of the healing abdominal wall tissue.

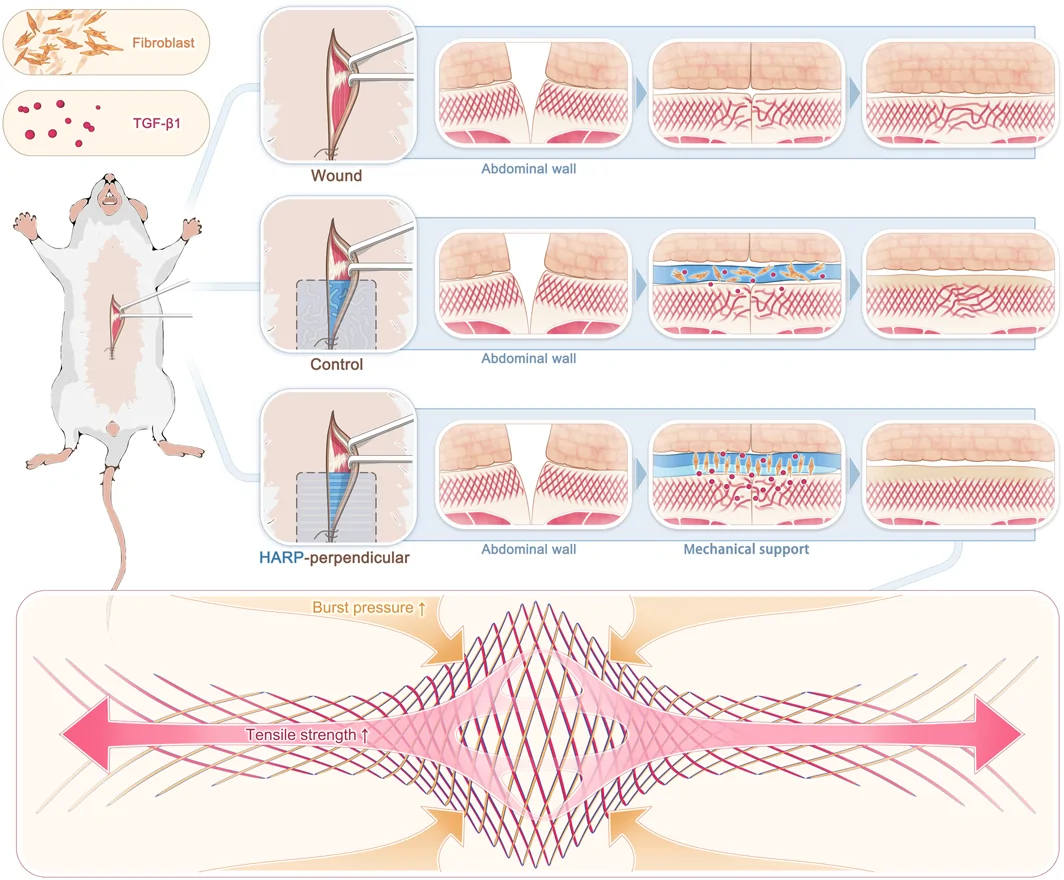

## 1 Introduction

The abdominal wall is a complex multi‐layered structure whose mechanical integrity is essential for physiological function [1]. Common surgical procedures, such as midline laparotomies, require incisions that compromise the linea alba, a critical load‐bearing connective tissue. This frequently leads to incisional hernias, one of the most common and severe complications of abdominal surgery, with incidence rates reported to be as high as 30% [2]. These hernias rarely heal spontaneously and typically require subsequent surgical intervention, causing significant patient morbidity and contributing to a tremendous financial burden on global healthcare systems [3].

The clinical gold standard for hernia repair relies on implanting surgical meshes, a strategy that is fundamentally reactive and addresses the mechanical defect without restoring native tissue function [4]. The most common materials, synthetic polymer meshes made of polypropylene, provide robust mechanical support but act as permanent foreign bodies [5]. These non‐resorbable meshes frequently lead to a cascade of clinical complications, including chronic inflammation, foreign body response, tissue adhesion, and a high risk of infection [6]. In an attempt to improve biocompatibility, biological grafts derived from decellularized animal tissues were introduced [7]. However, large‐scale clinical data have revealed these costly materials to be a suboptimal solution, associated with inconsistent degradation, immunogenic responses, and disappointingly high rates of hernia recurrence that can exceed those of synthetic meshes [8].

To address these material failures, a critical need exists for a new class of biomaterial that moves beyond simple mechanical reinforcement or passive replacement to actively guide the regeneration of functional load‐bearing tissue, thereby promoting early functional repair immediately following surgery. Hydrogel‐based biomaterials have emerged as a promising platform for this strategy, with research largely focused on enhancing their intrinsic material properties [9, 10]. This has led to the development of innovative “tough adhesives” with exceptional mechanical strength and tissue adhesion, as well as hydrogels with advanced antimicrobial functions [11, 12, 13, 14, 15, 16, 17, 18]. However, despite these significant advances in material chemistry, a fundamental limitation persists: these hydrogels are overwhelmingly isotropic materials [19]. They lack the instructive architectural cues necessary to overcome the default biological process of disorganized scar formation [20, 21]. While they can support healing, they cannot actively guide the anisotropic arrangement of new collagen fibers required to regenerate the highly organized and load‐bearing structure of the native linea alba [22].

We hypothesized that the therapeutic efficacy of a regenerative patch is influenced by the anisotropic orientation of its guiding architecture. To rigorously test this, we developed a Hydrogel‐based Architecture‐guided Regenerative Patch (HARP) featuring 3D‐bioprinted, parallel‐aligned, cell‐laden filaments oriented perpendicular to the incision that serve as a guiding template to promote organized, functional tissue regeneration at the defect site (Figure 1). We then directly compared its performance in a rat abdominal incision model to an identical construct with parallel orientation to the incision, thereby isolating the functional role of architectural guidance. To fabricate this structure, we first developed and characterized a novel gelatin/dialdehyde cellulose (DAC) bioink. We then demonstrated in vitro that this bioink, supplemented with TGF‐β1, promotes the desired fibroblast behaviors. Finally, we evaluated the therapeutic efficacy of HARP in vivo, showing that its biomimetic cell‐guiding architecture contributes to accelerated healing and restoration of abdominal wall mechanical strength.

**FIGURE 1:**
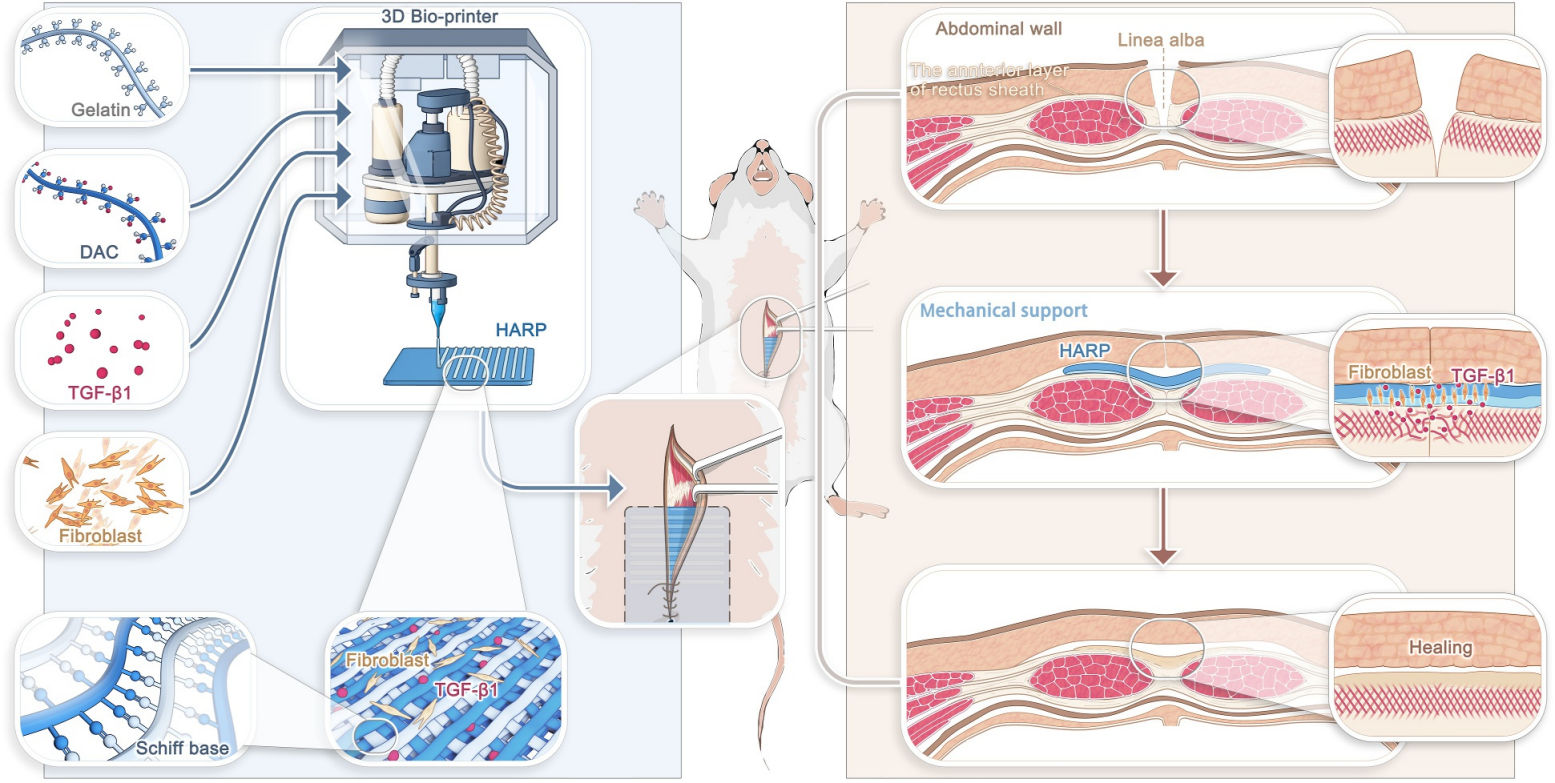
Schematic illustration of the 3D‐bioprinted HARP with perpendicularly aligned cell‐laden filaments that guide organized tissue regeneration at the incision site.

## 2 Results and Discussion

### 2.1 Surgical Incision Impairs the Mechanical and Microstructural Integrity of the Abdominal Wall

The high incidence of incisional hernias remains a major clinical challenge, stemming from the natural healing process that replaces strong, organized native tissue with mechanically weak, disorganized scar tissue [23]. To systematically elucidate the functional and microstructural impairments induced by a standardized midline laparotomy, rats underwent a midline laparotomy and were allowed to heal for 14 days (Wound group) before comparison to unoperated controls (Figure 2a). The incision profoundly compromised the mechanical strength of the abdominal wall (Figure 2b,c). Specifically, the Wound group exhibited a 67.0% reduction in tensile strength (2.96 ± 0.36 kN/m vs. 8.97 ± 1.06 kN/m for control) and a 59.4% reduction in burst pressure (68.07 ± 6.17 kPa vs. 167.60 ± 11.23 kPa for control).

**FIGURE 2:**
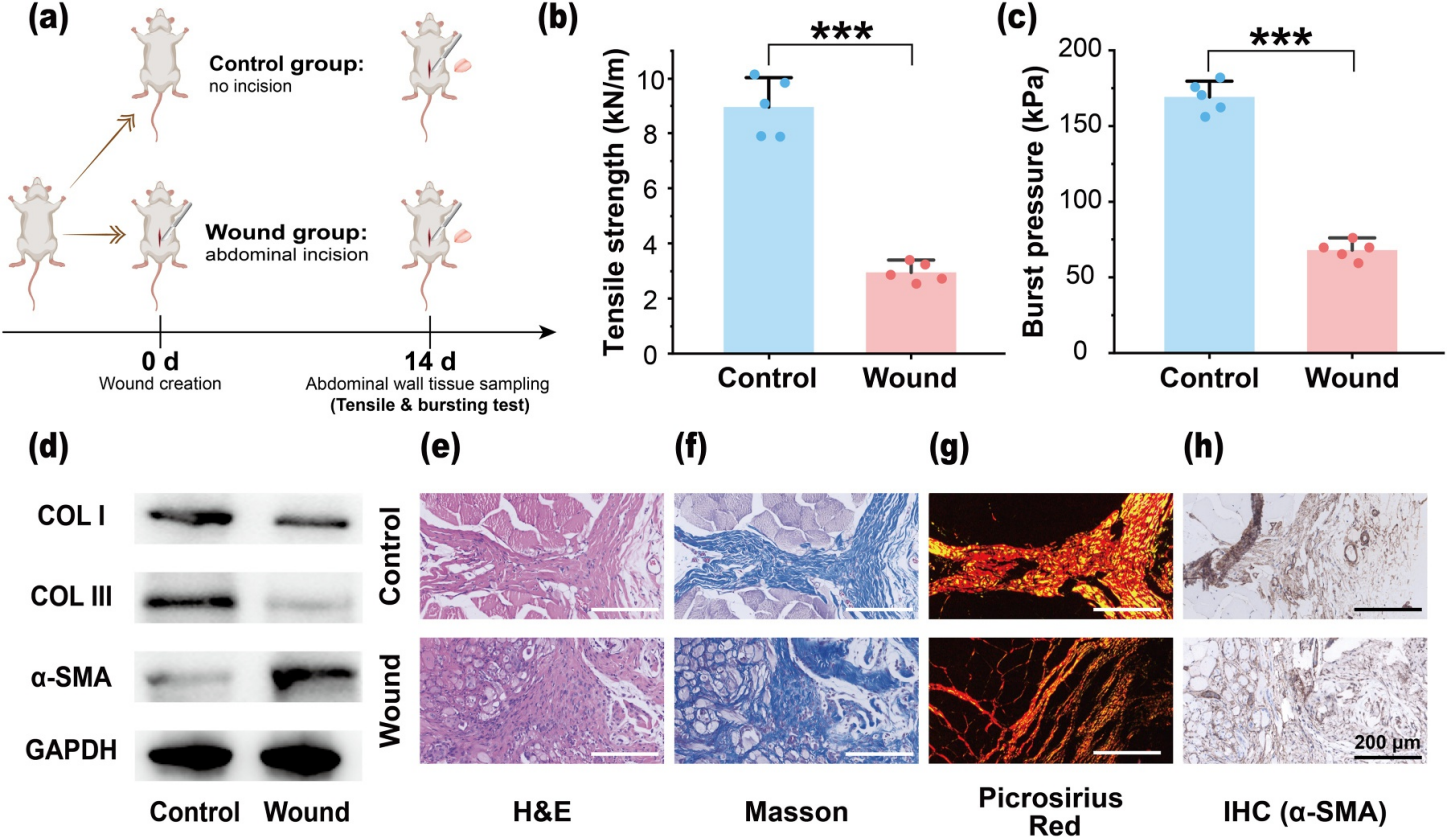
Surgical incision impairs the mechanical and microstructural integrity of the abdominal wall. (a) Schematic of the in vivo experimental design comparing the unoperated Control group to the incised Wound group over 14 days. (b) Tensile strength and (c) Burst pressure of the abdominal wall at day 14, both of which are significantly decreased in the Wound group. (d) Representative Western blot analysis showing reduced expression of Collagen I (Col I) and Collagen III (Col III) and elevated expression of α‐smooth muscle actin (α‐SMA) in wounded tissue. (e) H&E staining revealing disorganized tissue structure in the wound. (f) Masson's trichrome staining showing reduced and fragmented collagen fibers (blue) at the incision site. (g) Picrosirius Red staining under polarized light showing a marked reduction in densely organized yellow‐red birefringent collagen fibers and a sparse irregular collagen network at the incision site. (h) Immunohistochemistry confirming robust α‐SMA expression in the wound. Data in (b) and (c) are presented as mean ± SD (*n* = 5). Statistical significance was determined using a two‐tailed unpaired Student's t‐test. ***p < 0.001. Scale bars = 200 μm.

Microstructural and molecular analyses revealed the underlying cause of this mechanical failure. Western blot analysis showed that the expression of Collagen I (Col I) and Collagen III (Col III) was significantly decreased at the incision site, whereas the myofibroblast marker α‐smooth muscle actin (α‐SMA) was elevated (Figure 2d). This molecular signature indicates impaired ECM synthesis coupled with a persistent, unguided fibrotic response [24]. Histological evaluations corroborated these findings. H&E staining showed disorganized tissue and a disordered cellular arrangement (Figure 2e), while Masson's trichrome staining revealed reduced and discontinuous collagen fibers (Figure 2f). Picrosirius Red staining under polarized light revealed a marked reduction in densely organized yellow‐red collagen fibers, accompanied by a sparse and irregular collagen network at the incision site (Figure 2g). Robust α‐SMA expression, consistent with myofibroblast‐associated remodeling activity, was confirmed by immunohistochemistry (Figure 2h). Collectively, these data suggest that the natural healing process produces a structurally immature scar characterized by a disorganized, immature ECM that may be mechanically vulnerable.

### 2.2 Development and Characterization of a G_x_D_y_ Bioink

Based on the hypothesis that disorganized ECM remodeling is the root cause of incisional weakness, we developed a 3D‐bioprinted HARP to provide architectural guidance. The foundation of the HARP is a novel bioink composed of gelatin and dialdehyde cellulose (DAC) hydrogel (G_x_D_y_). Our choice of chemistry was deliberately oriented toward clinical translation, circumventing the drawbacks of the most common bioink, methacrylated gelatin (GelMA), which requires photo‐crosslinking with UV light and potentially cytotoxic photoinitiators [25, 26, 27]. Instead, our G_x_D_y_ system is based on a photoinitiator‐free and UV‐free Schiff base reaction, a type of dynamic covalent chemistry that proceeds spontaneously under physiological conditions to form robust and highly cytocompatible hydrogels [28].

DAC was synthesized from microcrystalline cellulose (MCC) via periodate oxidation, which introduced reactive aldehyde groups (Figure 3a). The ^13^C NMR spectrum showed a characteristic downfield shift of the C2 and C3 signals, and FTIR spectroscopy revealed a distinct aldehyde peak at 1720–1740 cm^−1^ (Figure 3b,c). DAC also exhibited reduced thermal stability and a significant loss of crystallinity compared to MCC (Figure 3d and Supporting Information S1: Figure S1). Moreover, DAC exhibited a broader particle size distribution compared to MCC (Supporting Information S1: Figure S2) and a near‐neutral surface charge (−0.11 ± 5.37 mV for DAC vs. 15.24 ± 7.71 mV for MCC, *p* < 0.05) (Supporting Information S1: Figure S3). Thermogravimetric analysis (Figure 3d) demonstrated that DAC had a lower thermal stability, also suggesting a disruption of the crystalline structure and hydrogen‐bonding network [29]. While pristine cellulose is not biodegradable in humans, the periodate oxidation process disrupts its crystalline structure, significantly enhancing its degradability and making it a suitable component for a regenerative implant [30]. In addition, we systematically optimized the synthesis conditions, including temperature, duration, NaIO_4_‐to‐MCC molar ratio, and pH (Supporting Information S1: Table S1). Correlation analysis identified reaction time and temperature as the critical contributors to product yield (Figure 3e and Supporting Information S1: Figure S4). After systematically optimizing the synthesis conditions, a reaction time of 48 h at 25°C was selected for all subsequent experiments (Supporting Information S1: Figure S5).

**FIGURE 3:**
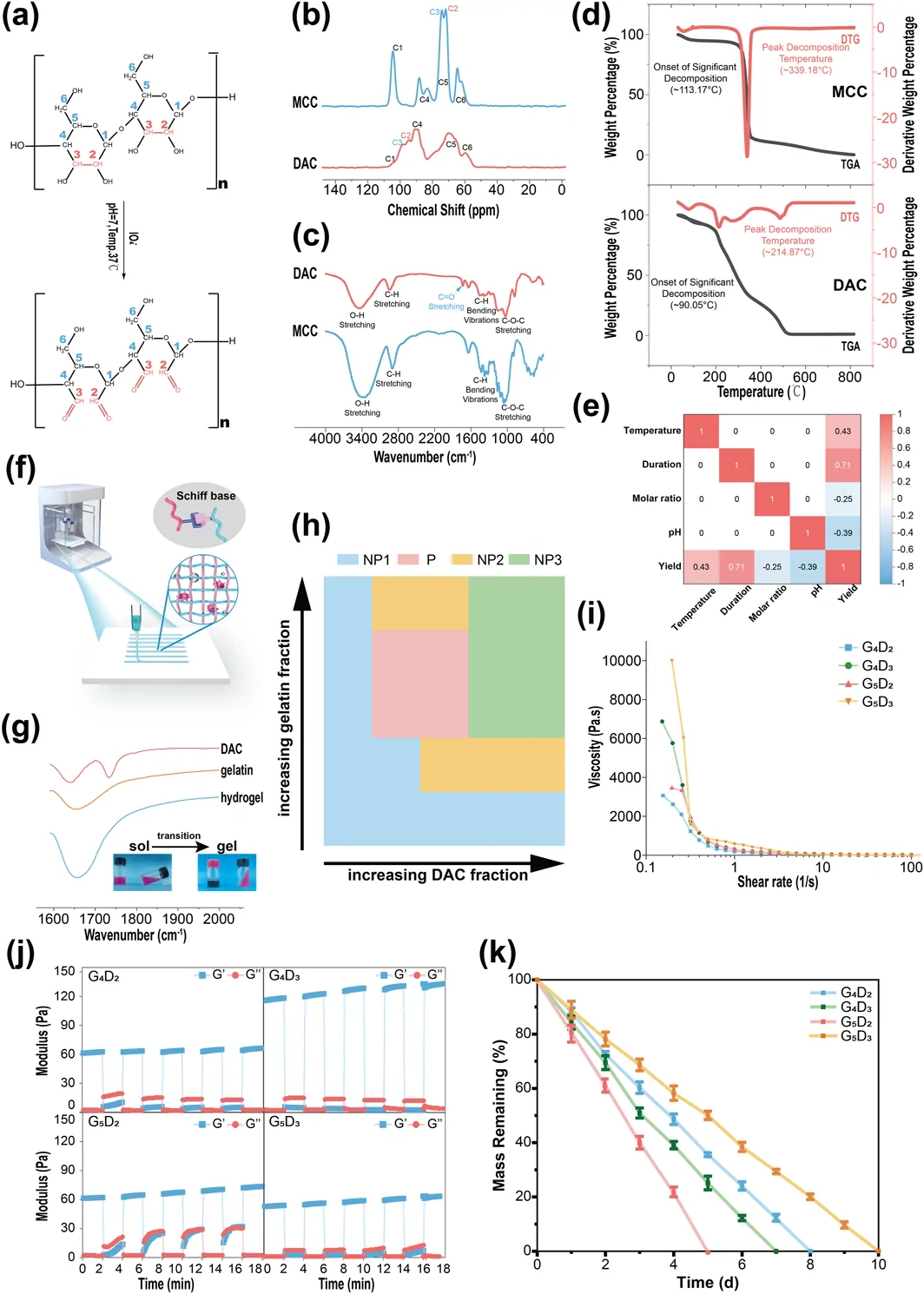
Synthesis, characterization, and optimization of the gelatin/dialdehyde cellulose (GxDy) bioink. (a) Schematic of DAC synthesis from microcrystalline cellulose (MCC) via periodate oxidation. (b) Solid‐state ^13^C NMR and (c) FTIR spectra confirming successful oxidation, indicated by the characteristic downfield shift of C2/C3 signals and the appearance of a new aldehyde peak (1720–1740 cm^−1^), respectively. (d) Thermogravimetric analysis (TGA/DTG) showing the reduced thermal stability of DAC compared to MCC. (e) Correlation analysis identifying reaction time and temperature as key determinants of product yield. (f) Schematic of hydrogel formation via Schiff base crosslinking between gelatin and DAC. (g) FTIR analysis confirming the formation of imine (C=N) bonds during the sol‐to‐gel transition. (h) Screening matrix identifying four lead candidate formulations (G_4_D_2_, G_4_D_3_, G_5_D_2_, G_5_D_3_) with optimal gelation kinetics for bioprinting. (i) Rheological analysis demonstrating the shear‐thinning behavior of the lead candidates. (j) Shear‐recovery tests showing robust and rapid recovery of the storage modulus (G′) after cyclic stress. (k) In vivo degradation profiles of the four candidates over 10 days. G_4_D_3_ formulation was selected for subsequent HARP fabrication due to its favorable balance of stability and degradation. Data are presented as mean ± SD (*n* = 3).

Our G_x_D_y_ system addresses a central challenge in 3D bioprinting: achieving high printability without compromising the construct's stability [26, 31]. While a recent advanced strategy involves using temporary small molecule modulators to dynamically tune crosslinking kinetics during extrusion, our study took an alternative approach by optimizing the intrinsic properties of the bioink itself [31]. The sol‐gel transition takes place via a spontaneous Schiff base reaction between the primary amine groups on gelatin and the aldehyde groups on DAC (Figure 3f), a process confirmed by FTIR analysis (Figure 3g). To optimize the formulation for bioprinting, 25 G_x_D_y_ formulations were screened based on their sol‐gel transition times (Figure 3h). Four lead candidates (G_4_D_2_, G_4_D_3_, G_5_D_2_, G_5_D_3_) exhibited suitable gelation kinetics for extrusion (Supporting Information S1: Figures S6 and S7). Rheological analysis confirmed that all four formulations possessed ideal properties for bioprinting, including pronounced shear‐thinning behavior and robust self‐recovery after stress (Figure 3i,j and Supporting Information S1: Figure S8).

Moreover, it was found that compressive modulus increased with crosslinking density (Supporting Information S1: Figure S9). This provides a wide range of tunable mechanical properties simply by altering component concentrations, offering a significant advantage for future studies investigating the role of mechanobiology in tissue regeneration [32]. Finally, in vivo degradation kinetics was evaluated via subcutaneous implantation (Figure 3k). The G_4_D_3_ formulation demonstrated an optimal balance between structural stability and a degradation rate suitable for tissue regeneration and was therefore selected for all subsequent HARP fabrication.

### 2.3 The Biocompatibility of G_4_D_3_ Bioink

The biocompatibility of the optimized G_4_D_3_ bioink was systematically evaluated. In vitro assays using fibroblasts showed high cell viability (98.71% ± 0.25% for bioink vs. 98.29% ± 0.49% for control) with no evidence of cytotoxicity after 72 h of exposure to bioink extracts (Figure 4a–c). Moreover, the bioink did not impair essential cellular functions, as fibroblast migration and wound closure rates were comparable to controls (e.g., the migration rate after 24 h, 40.1% ± 1.8% for control vs. 41.1% ± 1.1% for G_4_D_3_ bioink (Figure 4d, Supporting Information S1: Figures S10 and S11)). Crucially, the 3D bioprinting extrusion process did not compromise cell viability, with no significant difference in cell survival before and after printing (Figure 4e,f). The G_4_D_3_ hydrogel also demonstrated excellent hemocompatibility (Figure 4g), with a low hemolysis rate of 2.10% ± 0.15% (vs. 0.01% ± 0.004% for control), which was substantially lower than the positive control (99.94% ± 0.06%). Finally, in vivo safety was evaluated 14 days after subcutaneous implantation in rats. H&E staining of major organs (heart, liver, spleen, lungs, and kidneys) revealed intact tissue architecture with no obvious inflammatory cell infiltration or histopathological abnormalities (Figure 4h).

**FIGURE 4:**
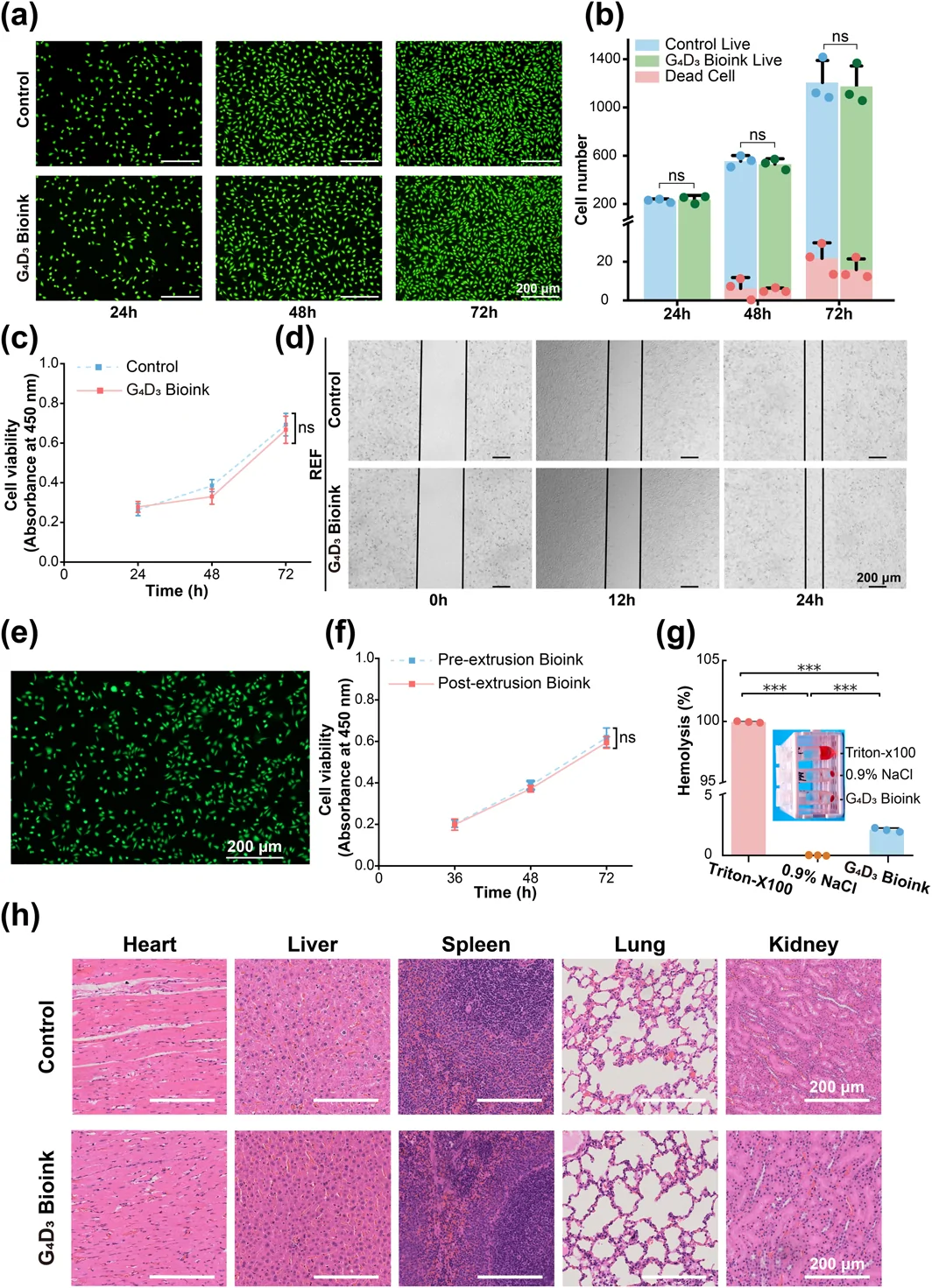
The optimized G_4_D_3_ bioink demonstrates excellent cytocompatibility and in vivo biocompatibility. (a) Representative Live/Dead fluorescence microscopy images and (b) quantification of fibroblasts cultured for up to 72 h with G_4_D_3_ bioink extract, showing high cell viability. (c) CCK‐8 assay confirming no significant difference in cell viability between the G_4_D_3_ and control groups over 72 h. (d) Scratch wound‐healing assay showing comparable migration and wound closure after 24 h. (e) Live/Dead staining and (f) CCK‐8 assay of encapsulated fibroblasts, showing that the 3D bioprinting extrusion process does not compromise cell viability. (g) Hemolysis assay showing a low hemolysis rate of the G_4_D_3_ bioink. (h) Representative H&E stained sections of major organs harvested 14 days after subcutaneous implantation, revealing intact tissue architecture with no signs of inflammation or damage. Data in (b), (c), (f), and (g) are presented as mean ± SD (*n* = 3). Statistical significance was determined using a two‐tailed unpaired Student's t‐test. ns, not significant; ***p < 0.001. Scale bars: 200 μm in (a, d, e, and h).

### 2.4 Formulation and Functional Characterization of the Cell‐Laden G_4_D_3_ Bioink

A key therapeutic principle of the HARP is to use its aligned cell‐laden structure as a template to guide the recruitment and organization of cells during wound repair. Therefore, the selection of the cellular and biochemical cues embedded within the bioink is critical. To make an informed choice, we used a bioinformatics re‐analysis of a public single‐cell RNA sequencing dataset as a hypothesis‐generating tool, which identified fibroblasts as central cellular players and TGF‐β1 as a key signaling molecule driving their matrix‐remodeling activity [33]. This analysis confirmed that fibroblasts are a dominant and dynamic cell population during repair, becoming central communication hubs by day 7 (Figure 5a,b) [34]. Ligand‐receptor analysis identified TGF‐β signaling, particularly TGF‐β1, as a key regulatory pathway driving fibroblast activity and differentiation into collagen‐secreting myofibroblasts (Figure 5c and Supporting Information S1: Figures S12‐S15) [35].

**FIGURE 5:**
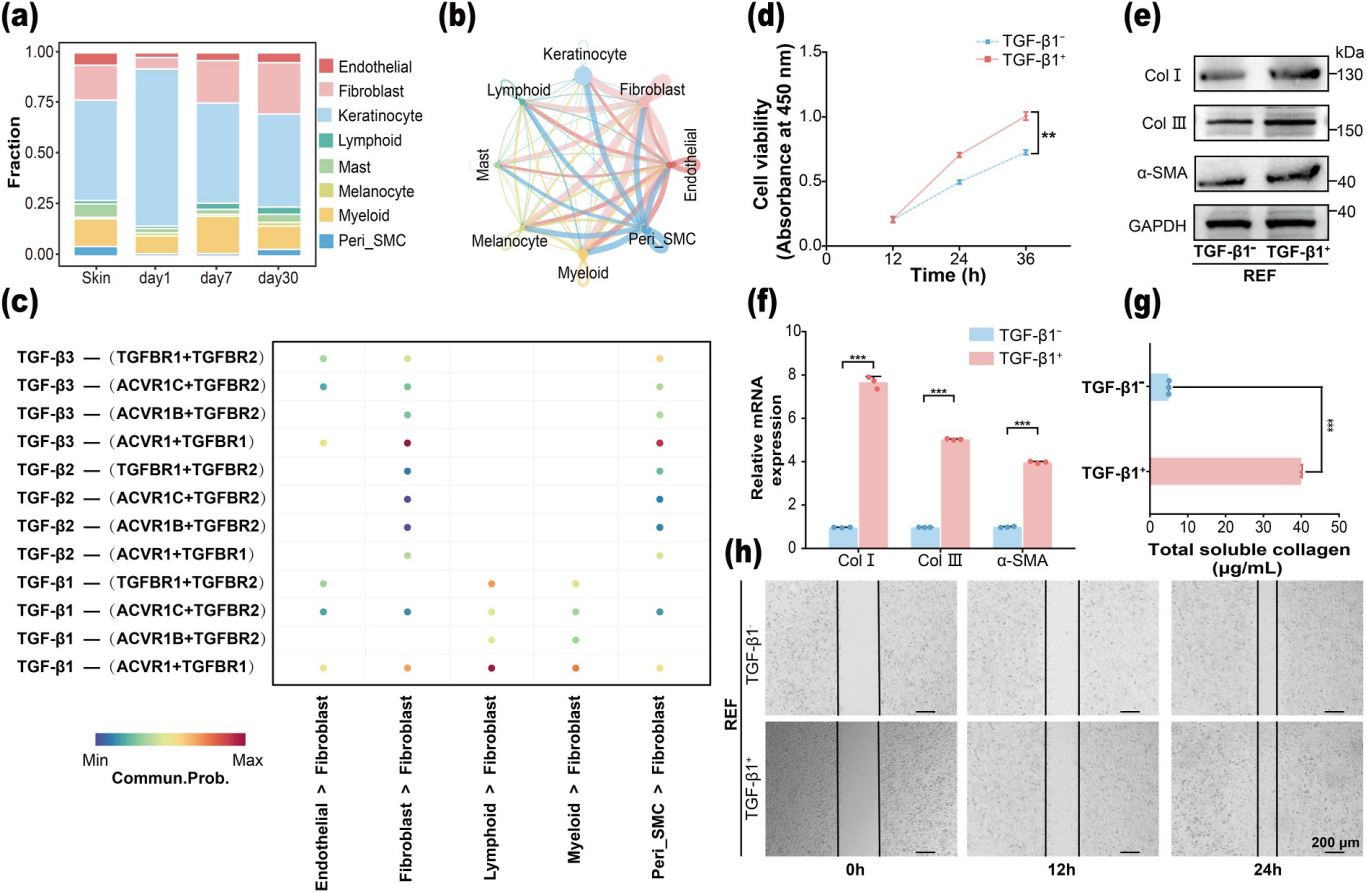
Bioinformatics rationale and in vitro validation of the fibroblast/TGF‐β1‐enriched bioink. (a–c) Bioinformatics re‐analysis of a public scRNA‐seq dataset from healing human skin wounds. (a) Cell population fractions over time, showing the dynamic expansion of fibroblasts. (b) Cell‐cell communication network at day 7, highlighting fibroblasts as a central hub. (c) Ligand‐receptor analysis identifying TGF‐β1 as a key signaling molecule interacting with fibroblasts. (d) Proliferation of rat embryonic fibroblasts (REFs) over 36 h with or without TGF‐β1, measured by CCK‐8 assay. (e) Representative Western blot and (f) qRT‐PCR analysis showing significant upregulation of Collagen I (Col I), Collagen III (Col III), and α‐SMA expression in REFs after TGF‐β1 treatment. (g) Quantification of total soluble collagen secreted by REFs after 48 h of treatment. (h) Representative images of a scratch wound healing assay demonstrating accelerated migration and wound closure in TGF‐β1‐treated REFs. Data in (d), (f), and (g) are presented as mean ± SD (*n* = 3). Statistical significance was determined using a two‐tailed unpaired Student's *t*‐test. **p < 0.01; ***p < 0.001. Scale bar in (h) = 200 μm.

While acknowledging the inherent biological differences between the dataset's source (human skin) and our model (rat fascia), we experimentally validated this rationale in vitro. As shown in Figure 5d, TGF‐β1 treatment significantly promoted fibroblast proliferation and markedly enhanced their migratory capacity in both scratch wound and Transwell assays (Figure 5h, Supporting Information S1: Figures S16 and S17). Furthermore, TGF‐β1 induced a potent pro‐fibrotic response, significantly upregulating the expression of key ECM proteins (Col I, Col III) and the myofibroblast marker α‐SMA at both the gene and protein levels (Figure 5e,f). This translated to a significant increase in total soluble collagen secretion (Figure 5g). Our results confirmed that TGF‐β1 potently promoted the desired pro‐regenerative behaviors in our rat fibroblast cell line, including proliferation, migration, and ECM protein synthesis [36, 37]. This evidence‐based approach justified our final bioink formulation of fibroblasts supplemented with TGF‐β1 to maximize the patch's regenerative potential.

### 2.5 HARP Accelerates Incisional Wound Healing and Restores Mechanical Strength *in Vivo*

Accelerated mechanical restoration may have translational relevance, as a shorter and more robust repair period can support earlier mobilization and potentially reduce postoperative complications associated with delayed wound healing. Unlike conventional meshes, which serve strictly as passive mechanical barriers, HARP is explicitly engineered as an active, bioinstructive template. To test the central hypothesis that architectural guidance contributes to functional repair, we conducted a rigorous in vivo study comparing four groups: untreated Wound, Isotropic Control hydrogel, HARP‐Parallel, and HARP‐Perpendicular (Figure 6a). The experimental setup for the burst‐pressure measurement is illustrated (Supporting Information S1: Figure S18). Mechanical testing revealed a stark divergence in outcomes based on architectural cues (Figure 6b). The Isotropic Control and HARP‐Parallel groups failed to provide a significant therapeutic benefit. Their tensile strengths (3.21 ± 0.65 kN/m for Isotropic Control and 2.85 ± 0.21 kN/m for HARP‐Parallel, respectively) were not significantly different from each other, and their burst pressures (79.40 ± 3.51 kPa for Isotropic Control and 62.33 ± 8.10 kPa for HARP‐Parallel, respectively) were comparable to, or in the case of the HARP‐Parallel group, even lower than the untreated Wound group (2.96 ± 0.36 kN/m for tensile and 68.07 ± 6.17 kPa for burst, respectively). In stark contrast, the HARP‐Perpendicular group demonstrated a dramatic and superior restoration of mechanical function, with a tensile strength of 7.00 ± 1.46 kN/m and a burst pressure of 132.26 ± 2.01 kPa. Its tensile strength was more than twice that of all other groups, while its burst pressure was substantially higher. These findings support the conclusion that filament orientation perpendicular to the incision contributes to functional repair [38]. The failure of the parallel‐aligned HARP to improve mechanical strength beyond the isotropic control suggests that the mere presence of aligned filaments is insufficient [23, 39, 40].

**FIGURE 6:**
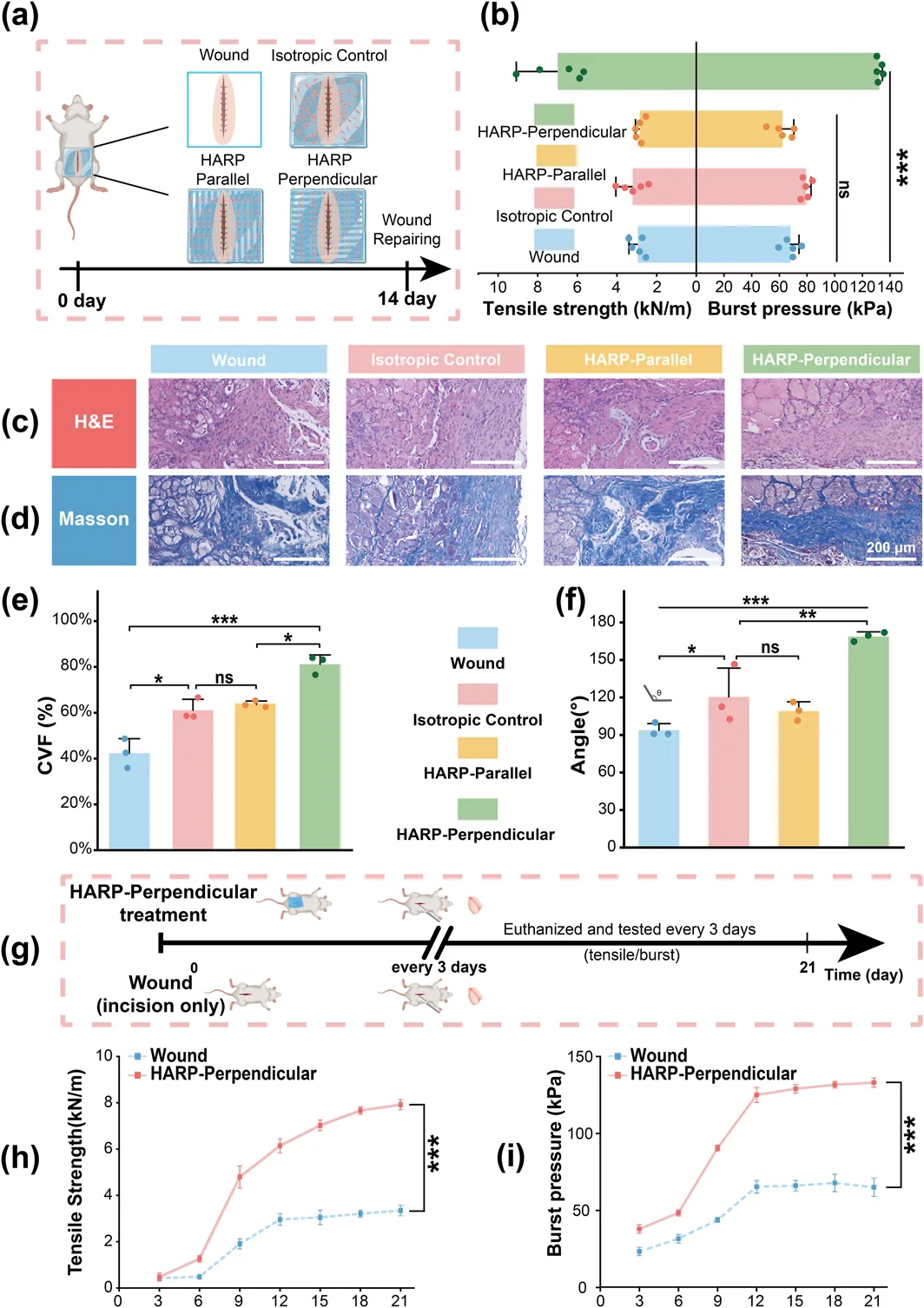
The HARP platform promotes oriented regeneration and accelerated mechanical restoration. (a) Schematic of the in vivo study design comparing four groups: untreated Wound, isotropic hydrogel (Isotropic Control), HARP with fibers parallel to the incision (HARP‐Parallel), and HARP with fibers perpendicular to the incision (HARP‐Perpendicular). (b) Tensile strength and burst pressure of the healed tissue at day 14. (c, d) Representative H&E and Masson's trichrome staining images showing a dense, well‐aligned collagenous matrix (blue) bridging the defect in the HARP‐Perpendicular group, in contrast to the disorganized scar‐like tissue in the other groups. (e) Quantification of the Collagen Volume Fraction (CVF) and (f) collagen fiber alignment (Angle), confirming the highest collagen content and greatest anisotropy (angles approaching 180°) in the HARP‐Perpendicular group. (g) Schematic of the temporal study design. (h,i) Temporal evaluation of tensile strength and burst pressure for 21 days. Data are presented as mean ± SD (*n* = 5 for mechanical tests and *n* = 3 for histological and quantitative analyses). Statistical significance was determined by one‐way ANOVA with Tukey's post hoc test or a two‐tailed unpaired Student's t‐test. ns, not significant; *p < 0.05, **p < 0.01; ***p < 0.001. Scale bars = 200 μm.

To understand the molecular basis of these mechanical differences, we analyzed protein expression in the healed tissues (Supporting Information S1: Figure S19). Western blot analysis revealed that both the poorly‐performing isotropic Control and the high‐performing HARP‐Perpendicular groups exhibited similarly elevated expression of Col I, Col III, and α‐SMA compared to the untreated Wound group. Histological analysis further supported the observed differences in tissue organization. H&E and Masson's trichrome staining revealed that while the Wound and Control groups healed with a characteristic disorganized scar‐like matrix, the HARP‐Perpendicular group regenerated tissue with dense, thick, highly organized collagen fibers aligned perpendicular to the incision axis, effectively bridging the defect (Figure 6c–f). Under polarized light, Picrosirius Red staining (Supporting Information S1: Figure S20) showed that the HARP‐Perpendicular group exhibited the densest and most highly organized birefringent collagen network, whereas the other groups displayed sparser and more disordered collagen fibers. Consistent with this high‐quality matrix deposition, robust α‐SMA expression was observed in the HARP‐Perpendicular group, consistent with sustained myofibroblast activity (Supporting Information S1: Figure S21).

To further assess the respective contributions of the bioink components and structural guidance, we performed in vivo component‐control experiments utilizing patches with parallel‐aligned filaments oriented perpendicular to the incision. Sub‐groups comprising the bare crosslinked matrix (Hydrogel), matrix with growth factor (Hydrogel + TGF‐β1), and matrix with cells (Hydrogel + Fibroblasts) were evaluated against the complete HARP construct in this perpendicular orientation via comprehensive histological and immunohistochemical analyses (Supporting Information S1: Figures S22–S25). While the Hydrogel and Hydrogel + TGF‐β1 groups exhibited limited tissue organization, the Hydrogel + Fibroblasts cohort demonstrated a partial increase in localized matrix deposition. Crucially, however, none of these partial formulations could reproduce the dense, highly organized and anisotropically aligned collagen networks achieved by the complete HARP system. These findings support a coordinated contribution of TGF‐β1, fibroblasts, and perpendicular architectural cues to the improved tissue organization observed with the complete HARP construct.

Finally, a temporal study using the optimal HARP‐Perpendicular design confirmed that it not only improved the quality of repair but also significantly accelerated the restoration of mechanical function (Figure 6g–i). By day 9, the HARP‐treated group had already achieved a tensile strength (4.80 ± 0.48 kN/m) that surpassed the maximum strength the Wound group would achieve over the entire 21‐day study (3.35 ± 0.22 kN/m). The HARP‐treated tissue continued to strengthen, reaching a final tensile strength of 7.92 ± 0.22 kN/m. A similar trend was observed for burst pressure, with the HARP group reaching a final burst pressure (133.04 ± 2.98 kPa) nearly double that of the Wound group (65.04 ± 5.87 kPa). This acceleration has potential clinical relevance. A shorter, more robust healing period could enable earlier patient mobilization and rehabilitation, thereby potentially reducing the risk of common post‐surgical complications like infection and adhesion formation, which are major drivers for the development of new therapeutic patches [13, 16].

To elucidate the molecular mechanisms underlying HARP‐mediated anisotropic repair, we performed bulk RNA‐seq on regenerating abdominal wall tissues. Bulk RNA‐seq of regenerating tissues revealed a distinct transcriptional profile associated with HARP treatment (Figure 7a,b, Supporting Information S1: Figures S26 and S27), characterized by the specific activation of mechanotransductive and matrix‐orchestration cascades. Compared to the chaotic cellular migration typical of unguided scar formation, HARP‐treated tissues exhibited marked upregulation of focal adhesion and mechanosensing mediators (e.g., *Fn1, Itgb3, Src*; Figure 7c) [41]. Gene Set Enrichment Analysis (Figure 7d–f) corroborated this finding, revealing robust enrichment in integrin‐mediated signaling, regulation of the actin cytoskeleton, and collagen fibril organization. Biologically, these findings suggest that the patch's parallel filaments, applied perpendicular to the incision, act as a bioinstructive template. The transcriptional changes are consistent with enhanced cell–matrix interactions, cellular adhesion, and cytoskeletal regulation. Consistent with this interpretation, HARP‐treated tissues showed upregulation of matrix‐remodeling genes (*Col1a1, Col3a1, Mmp14, and Sfrp2*) and enrichment of programs related to extracellular matrix remodeling, cell adhesion, cytoskeletal regulation, wound healing, and immune responses, whereas downregulated genes were associated with mitochondrial respiration, oxidative phosphorylation, energy metabolism, and muscle‐related programs (Figure 7b,c and Supporting Information S1: Figures S28–S32) [42]. Together with our in vivo component controls, these data support a model in which HARP integrates biological cues with structural guidance to promote the deposition of an organized load‐bearing fascial matrix.

**FIGURE 7:**
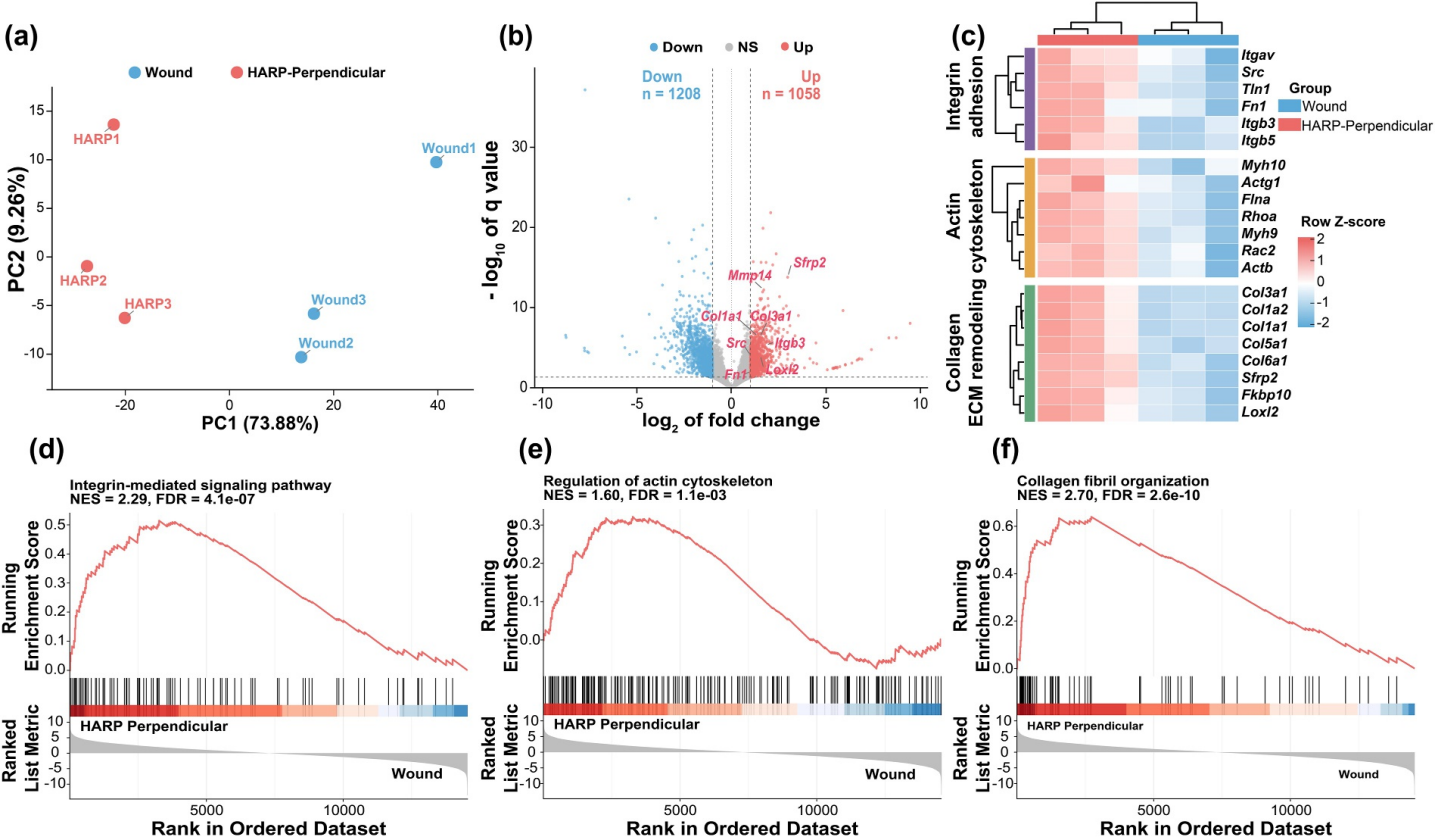
Transcriptomic analysis reveals that HARP‐Perpendicular activates adhesion‐, cytoskeleton‐, and collagen remodeling‐related repair programs. (a) Principal component analysis (PCA) of bulk RNA‐seq profiles from wound tissues in the Wound and HARP‐Perpendicular groups. Each point represents one biological replicate (*n* = 3 per group). (b) Volcano plot showing differentially expressed genes between the HARP‐Perpendicular and Wound groups. Red and blue dots indicate genes significantly upregulated and downregulated in the HARP‐Perpendicular group, respectively, while gray dots indicate non‐significant genes. Representative genes associated with ECM remodeling and cell‐matrix interactions, including Sfrp2, Mmp14, Col1a1, Col3a1, Itgb3, Src, and Fn1, are labeled. (c) Heatmap of selected mechanism‐related genes involved in integrin/adhesion signaling, actin cytoskeleton regulation, and collagen/ECM remodeling. Expression values are shown as row Z‐scores. (d–f) Gene set enrichment analysis (GSEA) showing significant enrichment of the integrin‐mediated signaling pathway (d), regulation of actin cytoskeleton (e), and collagen fibril organization (f) in the HARP‐Perpendicular group. NES, normalized enrichment score.

While this study provides a proof‐of‐concept for architecture‐guided fascial repair, several limitations remain. First, because this short‐term model evaluated early‐stage mechanical restoration, it does not directly capture long‐term hernia formation. Definitive validation of the capacity of HARP to mitigate herniation risk will require extended observation in dedicated large‐animal incisional hernia models. Second, although our data link architectural guidance to anisotropic remodeling, future studies employing spatial transcriptomics and in vivo cell‐tracking are needed to map the exact single‐cell dynamics driving this process. Finally, the reliance on exogenous fibroblasts introduces recognized manufacturing, storage, and regulatory hurdles. Future iterations will therefore explore an “off‐the‐shelf” acellular platform utilizing cell‐free cues such as exosomes. Despite these translational challenges, the profound functional restoration achieved through programmable architectural guidance strongly supports HARP's continued development as a next‐generation surgical patch.

## 3 Conclusion

In this study, we developed a novel Hydrogel‐based Architecture‐guided Regenerative Patch (HARP) featuring 3D‐bioprinted, parallel‐aligned filaments to actively support functional tissue repair at abdominal incision sites. When applied with these filaments oriented perpendicular to the incision, HARP guided the deposition of a dense anisotropic extracellular matrix that closely biomimicked native linea alba architecture. This structurally programmed remodeling yielded a more than twofold increase in mechanical strength and significantly accelerated the healing timeline compared to untreated wounds. Collectively, this work establishes a robust proof‐of‐concept for an architecture‐guided regenerative strategy that enhances fascial repair and may mitigate the risk of incisional herniation. Future preclinical evaluations in dedicated large‐animal models with extended follow‐up will be essential to confirm whether these early structural benefits translate into a long‐term reduction in hernia incidence.

## 4 Experimental Section

The experimental details are provided in the Supporting Information.

## Author Contributions

Tianxing Gong, Hao Li, and Xueqiang Peng conceived the study and designed the overall research framework. Hao Li, Xueqiang Peng, Xinwei Liu, Xinyan Zhang, and Qiushi Tang performed the experiments and conducted data analysis. Stephanie Willerth, Mario Taba Junior, and Ricardo M. Carvalho provided scientific advice on research direction, methodological choices, and interpretation of results. Tianxing Gong, Hailong Yu, Shibo Wei, and Hangyu Li supervised the project and provided funding support. Tianxing Gong, Hao Li, Xueqiang Peng, and Xinwei Liu drafted the manuscript, and all authors contributed to the review and final editing of the manuscript.

## Ethics Statement

All animal experiments were carried out strictly in accordance with the approved protocols (Approval No. 2025‐98, Northern Theater General Hospital Animal Medical Ethics Committee; Approval No. CMU20231483, Institutional Animal Care and Use Committee of China Medical University).

## Conflicts of Interest

Tianxing Gong is an inventor on a patent (No. 202310428437.4) related to the technology described in this work. All other authors declare no competing interests.

## Supporting information


Supporting Information S1

## Data Availability

The data that support the findings of this study are available on request from the corresponding author. The data are not publicly available due to privacy or ethical restrictions.
