# Supplementary material for: A 3D‐Bioprinted, Cell‐Guiding Hydrogel Patch Promotes Functional Repair of Abdominal Incisions via Anisotropic ECM Remodeling
Source: Smart Med. 2026 Sep 23;5(5):e70056. doi: 10.1002/smmd.70056 (PMC13618637; doi:10.1002/smmd.70056)
Supplement: Supplementary file 1 [file SMMD-5-e70056-s001.docx]

Supporting Information

A 3D-Bioprinted, Cell-Guiding Hydrogel Patch Promotes Functional Repair of Abdominal Incisions via Anisotropic ECM Remodeling

Tianxing Gong*, Hao Li, Xueqiang Peng, Xinwei Liu, Xinyan Zhang, Qiushi Tang, Stephanie Willerth, Mario Taba Junior, Ricardo M. Carvalho, Hailong Yu*, Shibo Wei*, Hangyu Li*

**Supplementary Material and Methods**

**1. Chemical and Reagents**

The main reagents used in this study included absolute ethanol (Fuyu Chemical, China), methanol (Kemio Chemical, China), xylene (Sigma-Aldrich, China), 0.9% sodium chloride solution (Kelun, China), 75% and 95% ethanol (Kangtai, China), povidone-iodine disinfectant (Lierkang, China), gelatin (from porcine skin, Type A; Sigma-Aldrich, China), and microcrystalline cellulose (Sigma-Aldrich, China). Buffers and media included 1× and 10× PBS (Procell, China), 1× TBST buffer (Biosharp, China), electrophoresis and transfer buffers (Servicebio/Yeasen, China), isoflurane (Shandong Antai Animal Husbandry Technology, China), and complete medium for rat embryonic fibroblasts (Procell, China). Fixatives and staining reagents comprised 4% paraformaldehyde, hematoxylin, eosin, neutral red, Sirius red, toluidine blue, crystal violet (all from Biosharp, China), and a Masson’s trichrome kit (Baso, China). For cell and molecular assays, reagents included trypsin-EDTA, serum-free cryopreservation solution (Procell, China), RIPA lysis buffer, EDTA (Biosharp, China), protease and phosphatase inhibitor cocktails (Yeasen, China), total RNA extraction kits (Takara Bio, China), RNase-free water (Takara Bio, China), fetal bovine serum (FBS; Sigma-Aldrich, China), and 5× SDS-PAGE loading buffer (Thermo Fisher, China). The rat embryonic fibroblast (REF) cell line was sourced from Procell Life Science & Technology (China). Commercial kits included the BCA Protein Assay Kit (Beyotime, China), Calcein/PI Cell Viability Kit (Beyotime, China), ECL Kit, CCK-8 Kit, DAB Substrate Kit (all from Biosharp, China), and the Fast Seven Total Protein Extraction Kit (Seven Biotech, China). Antibodies and proteins used were recombinant mouse/rat TGF-β1 (R&D Systems, China), primary antibodies against GAPDH, Col I, Col III, and α-SMA (Proteintech, China), a prestained protein marker (Abcam, China), and HRP-conjugated secondary antibodies (Abbkine, China).

**2. Determination of Aldehyde Content**

The aldehyde content of the synthesized DAC was quantified using the hydroxylamine hydrochloride titration method. Briefly, 0.2 g of dry DAC powder was dispersed in 25 mL of a hydroxylamine hydrochloride solution (0.25 mol/L, adjusted to pH 5.0). The suspension was stirred at 40 °C for 4 h. The hydrochloric acid (HCl) released was then titrated with a standardized 0.1 mol/L sodium hydroxide (NaOH) solution until the pH returned to 5.0. An identical titration was performed on MCC to serve as a blank control. The aldehyde content was calculated using the following equation:

Aldehyde Content (mmol/g) = (V_1_ − V_2_) × C/m

where V₁ and V₂ are the volumes (mL) of NaOH solution consumed for the DAC and MCC samples, respectively; C is the concentration (mol/L) of the NaOH solution; and m is the dry mass (g) of the DAC sample.

**3. 3D Bioprinting and Fidelity Assessment**

**3.1 3D Bioprinting Protocol**

The G_x_D_y_ hydrogel precursor solutions were loaded into a 5-mL syringe prior to complete gelation and installed in a 3D bioprinter. The hydrogels were printed into lattice scaffolds using the following standardized parameters: the cartridge temperature was maintained at 37 °C while the platform was at room temperature (~25 °C). Printing was performed through a 0.19-mm diameter nozzle at a speed of 8 mm/s with an air pressure of 0.08 MPa. The final scaffolds consisted of 15 layers with an infill distance of 1.5 mm and a retraction distance of 2 mm.

**3.2 Quantification of Printing Fidelity**

The shape fidelity of printed constructs was quantified to evaluate how closely they matched the original computer-aided design (CAD). Four lead hydrogel formulations were tested: G_4_D_2_, G_4_D_3_, G_5_D_2_, and G_5_D_3_. For each formulation, a grid structure was printed using the standardized parameters. Immediately after printing, the constructs were imaged using a digital camera (EOS 90D, Canon, Japan), and their actual dimensions were measured using ImageJ software.

Fidelity was assessed by calculating both the surface fidelity (Dₛ) and layer fidelity (Dₕ) using the following equations:

Surface Fidelity (D_s_) = S_1_/S_0_ × 100%

Layer Fidelity (D_h_)= H_1_/H_0_ × 100%

, where S₀ and S₁ represent the designed and measured surface areas, respectively, and H₀ and H₁ represent the designed and measured layer heights, respectively.

**4. Cell Viability (CCK-8 Assay)**

All viability and proliferation assays were conducted using a Cell Counting Kit-8. Absorbance was measured at 450 nm using a microplate reader (iMark™, Bio-Rad, U.S.A.). For all assays, hydrogel extracts were prepared by incubating sterilized hydrogel samples in rat embryonic fibroblast (REF) culture medium for 24 h at 37 °C, following ISO 10993-5 guidelines.

**4.1 Hydrogel Cytotoxicity**

To assess the hydrogel’s cytotoxicity, REFs were seeded into 96-well plates at a density of 5 × 10³ cells/well. After 24 h, the culture medium was replaced with either the standard G_4_D_3_ hydrogel extract (experimental group) or fresh medium (control group) and cultured for 24, 48, and 72 h. Viability was assessed at each time point using the standard CCK-8 protocol. There were 3 replicates in each group (n = 3).

**4.2 Post-Printing Viability**

To evaluate the effect of the bioprinting process, extracts were prepared from two types of G_4_D_3_ hydrogels: non-extruded hydrogel cast directly into a mold (pre-extrusion extract) and hydrogel that was first extruded through the 3D bioprinter nozzle (post-extrusion extract). These respective extracts were then applied to REF cultures, and viability was assessed after 72 h. There were 4 replicates in each group (n = 3).

**4.3 TGF-β1 Proliferation Assay**

To quantify the proliferative effect of TGF-β1, extracts were prepared from two groups of G_4_D_3_ hydrogels: a control hydrogel without TGF-β1 (control extract) and an experimental hydrogel formulated with TGF-β1 (TGF-β1-conditioned extract). REFs were seeded into 96-well plates at a density of 2 × 10³ cells/well and treated with the different extracts (three replicates per group). Proliferation was quantified at 12, 24 and 36 h using the standard CCK-8 protocol.

**5. Cell Migration Assays**

**5.1 Cell Scratch**

The migratory capacity of REFs was assessed using a scratch assay. REFs were seeded into 6-well plates at a density of 1 × 10^5^ cells/well and cultured in 2 mL of complete medium in a 37 °C, 5% CO₂ incubator (Midi 40, Thermo, U.S.A.) until a confluent monolayer formed. A linear scratch was created in the center of each well using a sterile 200-µL pipette tip. The monolayer was then washed twice with PBS to remove detached cells before the addition of the experimental media. Two separate experiments were conducted with this protocol. To assess the hydrogel's effect, cells were treated with either fresh culture medium (control group) or the G_4_D_3_ hydrogel extract. To validate the effect of TGF-β1, cells were treated with either culture medium without TGF-β1 (TGF-β1^−^) or medium supplemented with TGF-β1 (TGF-β1^+^).

Images of the scratch were captured at 0, 12, and 24 h using an inverted microscope (Nikon TS100, Nikon, Japan). The open area of the scratch was quantified using ImageJ software (NIH, U.S.A.), and the wound closure rate was calculated as follows:

Wound Closure Rate (%)= (Area_t=0_ – Area_t_)/Area_t=0_ × 100%

**5.2 Transwell Assay**

Cell migration was further quantified using 8-µm pore size Transwell inserts. Rat embryonic fibroblasts (REFs) were harvested and resuspended to a density of 5 × 10⁴ cells/mL in serum-free medium containing the specific experimental treatment. A 200-µL volume of the cell suspension was added to the upper chamber of the Transwell insert. The lower chamber was filled with 600 µL of complete medium containing 10% FBS, which served as a chemoattractant. After 24 h of incubation (37 °C, 5% CO₂), non-migrated cells remaining in the upper chamber were removed with a cotton swab. Cells that had migrated to the lower surface of the membrane were then washed with PBS, fixed with 4% paraformaldehyde for 20 min, and stained with 0.1% crystal violet for 15 min. After a final wash, the number of migrated cells was determined by counting cells in five random fields of view per insert under an inverted microscope and calculating the average.

This protocol was used to assess two conditions. To evaluate the effect of the hydrogel, cells in the upper chamber were suspended in either serum-free medium (control group) or G_4_D_3_ hydrogel extract (experimental group). To evaluate the effect of TGF-β1, cells were suspended in serum-free medium with (experimental group) or without (control group) TGF-β1.

**6 Hemolysis Assay**

The blood compatibility of the G_4_D_3_ hydrogel was evaluated using a hemolysis assay. Whole blood was collected from the abdominal aorta of isoflurane-anesthetized rats into EDTA-containing tubes. Red blood cells (RBCs) were isolated by centrifugation (3000 rpm, 15 min), washed three times with 0.9% saline until the supernatant was clear, and resuspended in saline to a 2% v/v solution.

For the assay, 500 µL of the RBC suspension was incubated at 37 °C for 1 h with one of the following: 500 µL of TritonX-100 (positive control), 500 µL of 0.9% saline (negative control), or 500 µL of G_4_D_3_ hydrogel extract (experimental group). After incubation, samples were centrifuged (2400 rpm, 5 min), and the absorbance of the supernatant, corresponding to released hemoglobin, was measured at 540 nm using the microplate reader (iMark™, Bio-Rad, U.S.A.). The hemolysis rate (%) was calculated as follows:

Hemolysis (%) = (Abs _Sample_ − Abs_Negative Control_) / (Abs_Positive Control_ −Abs_Negative Control_) × 100%

**7. Gene Expression Analysis (qRT-PCR)**

The effect of TGF-β1 on gene expression in REFs was assessed by quantitative real-time PCR (qRT-PCR). Total RNA was extracted from cell lysates using an RNeasy Mini Kit and reverse-transcribed into cDNA using a cDNA Reverse Transcription Kit, both according to the manufacturers' instructions.

qRT-PCR was performed using SYBR™ Green Master Mix on a CFX96™ Real-Time PCR System (Bio-Rad, U.S.A.) The reverse transcription reaction was performed at 52 °C for 300 s, followed by denaturation at 95 °C for 10 s. The qRT-PCR cycling conditions were as follows: denaturation at 95 °C for 5 s and annealing/extension at 60 °C for 30 s, repeated for 40 cycles. After amplification, C_t_ values of target and reference genes were obtained.

The relative mRNA expression of Col1a1, Col3a1, and Acta2 (α-SMA) was calculated from the threshold cycle (Ct) values using the 2^⁻ΔΔCt^ method, with Gapdh serving as the housekeeping gene for normalization. All experiments were performed in triplicate. Primer sequences are listed in Table S2.

**8. Western Blot Analysis**

Tissue or cell samples were lysed on ice for 30 min in ice-cold RIPA buffer supplemented with 1 mM PMSF and a phosphatase inhibitor cocktail. Tissue samples were first homogenized by grinding in liquid nitrogen, followed by sonication. Lysates were clarified by centrifugation at 12,000 × g for 15 min at 4 °C, and the protein-containing supernatant was collected. Total protein concentration was determined using a BCA Protein Assay Kit. Equal amounts of protein (20–40 µg per lane) were denatured at 95 °C for 5 min and separated on an 8–12% SDS-polyacrylamide gel. The separated proteins were then transferred to a 0.45-µm polyvinylidene fluoride (PVDF) membrane.

Membranes were blocked for 1 h at room temperature in 5% nonfat dry milk in TBST. After blocking, membranes were incubated overnight at 4 °C with the appropriate primary antibodies. Membranes were then washed three times for 10 min each in TBST and incubated for 2 h at room temperature with the appropriate HRP-conjugated secondary antibody. After final washes, protein bands were visualized using an ECL kit on a chemiluminescence imaging system (ChemiDoc™ XRS+, Bio-Rad, U.S.A.). Band intensities were quantified using ImageJ software and normalized to GAPDH.

**9. Soluble Collagen Production Assay**

To quantify collagen production, REFs were seeded in 24-well plates at a density of 3 × 10⁴ cells/well. After allowing the cells to adhere, the culture medium was replaced with fresh medium either with or without 10 ng/mL TGF-β1. Following a 48 h treatment period, the culture supernatant was collected from each well. The concentration of total soluble collagen in the supernatant was then quantified using a Sircol™ Soluble Collagen Assay kit according to the manufacturer’s instructions. Collagen content was calculated from a standard curve, and all experiments were performed in triplicate (n = 3).

**10. In Vivo Assessment of HARP**

**10.1 Animal Model and Ethics Statement**

Male Sprague–Dawley (SD) rats (6–8 weeks old) were obtained from SPF (Beijing) Biotechnology Co., Ltd., China. All animal experiments were carried out strictly in accordance with the approved protocols (Approval No. 2025-98, Northern Theater General Hospital Animal Medical Ethics Committee; Approval No. CMU20231483, Institutional Animal Care and Use Committee of China Medical University).

**10.2 Fabrication of the Hydrogel-based Architecture-guided Regenerative Patch (HARP)**

A 5% (w/v) gelatin solution was prepared to serve as the hydrogel matrix. A cell suspension of REFs (5 × 10⁶ cells per tube) was collected and centrifuged at 1000–1500 rpm for 5–10 min at room temperature. After discarding the supernatant, the cell pellet was resuspended and thoroughly mixed into the 5% gelatin solution. Subsequently, the DAC solution was added to the cell-gelatin suspension at the appropriate ratio to form the final G_4_D_3_ system, yielding the printable cell-laden bioink.

The HARP was fabricated using a two-step process. First, an acellular G_4_D_3_ hydrogel precursor solution was cast into a sterile mold (4 cm × 4 cm) and allowed to crosslink, forming a flexible substrate layer approximately 1.5 mm thick. This substrate was then secured to the platform of the 3D bioprinter. For the second step, the pre-formulated, cell-laden bioink was loaded into the printer and an aligned, filamentous structure was printed directly onto the surface of the substrate layer. The printing parameters were set as follows: layer height, 0.2 mm; printing speed, 8 mm/s; and line spacing, 600 µm. After printing was complete, the final composite patch was immediately transferred to a petri dish containing complete culture medium and pre-cultured for 24 h in a 37 °C, 5% CO₂ incubator for subsequent experiments.

**10.3 Surgical Procedure**

Rats were anesthetized with isoflurane and secured in a supine position. Following shaving and disinfection of the abdominal area with povidone-iodine, a 2 cm full-thickness incision was made along the midline (linea alba), beginning 1 cm below the xiphoid process, to expose the abdominal cavity. After the designated treatment was applied (e.g., placement of the HARP), the abdominal wall and skin were closed in successive layers using a 4-0 non-absorbable continuous suture.

**10.4 Experimental Groups and Study Design**

Multiple independent experiments were conducted, each with n=5 rats per group and a final observational endpoint of 14 days post-surgery. To establish the baseline effect of the incision, unoperated rats (Control) were compared to rats that received the surgical procedure (Wound). The primary therapeutic efficacy of the HARP's architecture was then evaluated by comparing the untreated wound group (Wound) to groups treated with either a non-aligned, cell (fibroblast/TGF-β1)-laden G_4_D_3_ hydrogel (Isotropic Control) or parallel-aligned HARP (HARP-Parallel), or a perpendicular-aligned HARP (HARP-Perpendicular).

**10.5 Tissue Harvesting and Processing**

At the designated endpoint, rats were euthanized by isoflurane overdose. For histological analysis, tissue from the incision site was harvested from all surgical groups. For the *in vivo* biocompatibility study, major organs (heart, liver, spleen, lungs, and kidneys) were also collected. All tissues intended for histology were fixed in 4% paraformaldehyde for 24 h, dehydrated through a graded ethanol series, cleared with xylene, and embedded in paraffin. Subsequently, 5-µm thick sections were prepared for staining. For mechanical testing, the entire anterior abdominal wall was excised.

**10.6 Histological and Immunohistochemical (IHC) Analysis**

For all staining, paraffin-embedded tissue sections (5 µm) were first deparaffinized in xylene and rehydrated through a graded ethanol series.

*Hematoxylin and Eosin (H&E) Staining*

For general morphological assessment, rehydrated sections were stained with hematoxylin for 3–5 min. Sections were then differentiated in 1% HCl-alcohol for 2–5 s and blued in Scott's tap water substitute for 30 s. Counterstaining was performed with eosin for 20 s – 4 min. Finally, sections were dehydrated, cleared, and mounted with neutral balsam for microscopic (BX53, Olympus, Japan) evaluation of tissue structure, collagen arrangement, and inflammatory cell infiltration.

*Masson's Trichrome Staining*

To specifically evaluate collagen deposition, rehydrated sections were stained with Weigert's iron hematoxylin for 5–10 min, followed by Biebrich Scarlet-Acid Fuchsin for 5–10 min. After differentiation in phosphomolybdic/phosphotungstic acid for 5–10 min, sections were directly stained with aniline blue for 5–10 min and briefly washed in 1% acetic acid. Following dehydration and clearing, sections were mounted. This protocol renders collagen fibers blue, muscle and cytoplasm red, and nuclei dark blue to black.

*Picrosirius Red Staining*

To assess collagen fiber type and organization, rehydrated sections were stained for 1 h in a saturated picric acid solution containing Picrosirius Red. After a brief differentiation in 0.5% acetic acid, sections were dehydrated, cleared, and mounted. The sections were then observed under a polarized light microscope (BX53, Olympus, Japan) , where type I collagen fibers appear yellow-red and type III collagen fibers appear green.

*Immunohistochemistry (IHC)*

For specific protein detection, sections first underwent antigen retrieval by microwave heating (10–15 min) in citrate buffer (pH 6.0). Endogenous peroxidase activity was blocked with 3% hydrogen peroxide for 10 min. After blocking non-specific binding with 5% BSA for 30 min, sections were incubated overnight at 4 °C with primary antibodies against Collagen I and Collagen III. On the following day, sections were incubated with an HRP-conjugated secondary antibody for 30 min at room temperature. The signal was developed using a DAB substrate, and nuclei were counterstained with hematoxylin. Stained tissue sections were observed and photographed using an upright light microscope .

**10.7 Mechanical Testing**

All mechanical tests were performed within 1 h of euthanasia.

*Tensile Strength Test*

The anterior abdominal wall was excised, sutures were removed, and four rectangular strips (20 × 5 mm), centered on the original incision, were prepared from each rat. Strips were kept in PBS until being mounted on a universal testing machine with the incision site centered. A preload of 0.1 N was applied, and each strip was pulled at a rate of 10 mm/min until failure. The maximum tensile force was recorded. To normalize tensile performance by specimen width, tensile strength per unit width was calculated as:

$$T \left( \mathrm{kN}/m \right)=F_{\left( \max\right)} \left( N \right)/\left[ w \left( m \right)\times1000 \right]$$

where T is the tensile strength per unit width (kN/m), F_max_ is the maximum tensile force (N), and w is the specimen width (m). All specimens had a width of 5 mm (0.005 m), and the normalized tensile values were expressed as kN/m.

*Burst Pressure Test*

Following euthanasia and suture removal, the entire anterior abdominal wall was excised and mounted on a lab-made burst pressure apparatus (Figure S18) with the original incision centered. Air pressure was gradually increased until the specimen ruptured, and the peak pressure value was recorded by a computer.

**10.8 *In Vivo* Degradation Study**

To assess the *in vivo* degradation kinetics, pre-weighed, lyophilized hydrogel discs (10 mm in diameter and 2.0 mm in thickness; initial mass denoted as W₀) were implanted subcutaneously on the dorsum of male Sprague-Dawley rats. The surgical site was prepared under isoflurane anesthesia by shaving and disinfection with povidone-iodine, and the incision was closed with sutures post-implantation.

At predetermined time points (n = 3 for each time point), animals were euthanized, and the remaining hydrogel implants were carefully explanted, lyophilized, and weighed (Wₜ). The degradation endpoint was defined as the time point at which the hydrogel was no longer macroscopically visible. The residual mass percentage was calculated as follows:

Residual Mass (%) = W_t_/W_o_ × 100%

***10.9 RNA Sequencing Analysis***

At day 14 after surgery, regenerating abdominal wall tissues encompassing the incision site were collected from the Wound and HARP-Perpendicular groups (n = 3 biological replicates per group). Total RNA was extracted using TRIzol reagent following the manufacturer’s protocol. RNA quality and integrity were assessed, and high-quality RNA samples were used for library construction. Following mRNA enrichment using oligo-dT beads, purified transcripts were fragmented and reverse-transcribed into cDNA. The libraries were then sequenced on an Illumina NovaSeq X Plus platform using a paired-end sequencing strategy.

Raw reads were filtered to remove adaptor sequences and low-quality reads, and clean reads were aligned to the rat reference genome using HISAT2. Gene expression levels were quantified using StringTie.

Downstream transcriptomic analyses were performed in R. Differential gene expression analysis between the Wound and HARP-Perpendicular groups was performed using DESeq2 with gene-level raw counts as input. Genes with an adjusted P value < 0.05 and |log2 fold change| ≥ 1 were considered differentially expressed genes. Sample-to-sample correlation analysis and principal component analysis (PCA) were performed based on normalized gene expression data. Heatmaps were generated using normalized expression values and displayed as row Z-scores.

Gene Ontology (GO) biological process enrichment analysis and Kyoto Encyclopedia of Genes and Genomes (KEGG) pathway enrichment analysis were performed for differentially expressed genes. Gene Set Enrichment Analysis (GSEA) was performed using ranked gene lists to identify repair-associated molecular pathways involved in HARP-mediated extracellular matrix remodeling.

**11. scRNA-seq Data Re-analysis**

A publicly available single-cell RNA sequencing (scRNA-seq) dataset of intact human skin and wound biopsies (days 1, 7, and 30 post-injury) was re-analyzed in R (v4.4.3) using the Seurat package (v4.3.0). After standard quality control filtering, data were log-normalized, and the top 2000 highly variable genes were identified. Confounding variance from mitochondrial gene content and cell cycle effects was regressed out during scaling.

Principal component analysis (PCA) was performed for dimensionality reduction. The resulting principal components were integrated using the Harmony algorithm to correct for batch effects across samples. The integrated data were used for Uniform Manifold Approximation and Projection (UMAP) visualization and graph-based clustering. Major cell populations were annotated using canonical marker genes. The identified fibroblast population was then computationally isolated and sub-clustered for a more refined analysis of heterogeneity. Cell-type distributions across time points were quantified and visualized. Intercellular communication networks were inferred using CellChat, and specific ligand-receptor interactions involving the TGF-β pathway directed at fibroblasts were visualized.

**Supplementary Figures**

**Figure S1. X-ray diffraction (XRD) analysis of microcrystalline cellulose (MCC) before and after oxidation.**

**Figure S2. Particle size distribution of MCC and DAC.**

**Figure S3. Zeta potential of MCC and DAC. Data are presented as mean ± SD (n=3). Statistical significance was determined by a two-tailed unpaired Student’s t-test (*p* < 0.05).**

**Figure S4. Correlation analysis of DAC synthesis parameters. A correlation matrix (heatmap) shows the relationships between key synthesis parameters (Temperature, Duration, Molar ratio, pH) and the final Recovery rate. The color and numerical value in each cell represent the Pearson correlation coefficient, where 1 (red) indicates a perfect positive correlation and -1 (blue) indicates a perfect negative correlation.**

**Figure S5. Optimization of DAC synthesis reaction time. Quantification of aldehyde content (Aldehyde Amt., left y-axis) and total recovery rate (right y-axis) as a function of reaction time for the periodate oxidation of MCC at 25 °C. Data are presented as mean ± SD (n=3).**

**Figure S6. Shape fidelity and stability of 3D-printed GxDy hydrogel scaffolds. Macroscopic images (side view, top row; top-down view, bottom row) of 3D-printed lattice scaffolds fabricated from the four lead candidate bioink formulations: G₄D₂, G₄D₃, G₅D₂, and G₅D₃. Scale bars = 2 mm.**

**Figure S7. Quantitative analysis of printing fidelity for the lead candidate GxDy formulations. The degree of conformity (%) was calculated for both the printed Area (surface fidelity) and Height (layer fidelity) relative to the original computer-aided design. All four formulations demonstrated a high degree of conformity, confirming their excellent printability and shape fidelity. Data are presented as mean ± SD (n=3).**

**Figure S8. Oscillatory amplitude sweep rheology of the lead candidate GxDy formulations. The storage modulus (G′, solid lines) and loss modulus (G′′, dashed lines) of the four lead candidate hydrogels (G₄D₂, G₄D₃, G₅D₂, and G₅D₃) as a function of increasing shear stress.**

**Figure S10. Effect of G₄D₃ bioink extract on fibroblast migration. Quantification of the relative migration area (wound closure rate) from a scratch wound healing assay after 24 hours of treatment with either control medium or G₄D₃ bioink extract. Data points represent individual replicates, and bars represent the mean ± SD (n=3). Statistical significance was determined by a two-tailed unpaired Student’s t-test; ns, not significant (*p* > 0.05).**

**### Summary of Key Improvements**

**Figure S9. Compressive modulus of the lead candidate GxDy hydrogels. Data are presented as mean ± SD (n=3). Statistical significance was determined by one-way ANOVA with Tukey's post hoc test. *p < 0.05.**

**Figure S11. Effect of G₄D₃ bioink extract on fibroblast migration in a Transwell assay. (Top) Representative microscopy images of crystal violet-stained fibroblasts that migrated through the Transwell membrane. (Bottom) Quantification of the number of migrated cells per field after 24 hours of treatment with either control medium or G₄D₃ bioink extract. Data points represent individual replicates, and bars represent the mean ± SD (n=3). Statistical significance was determined by a two-tailed unpaired Student’s t-test; ns, not significant (*p* > 0.05). Scale bars = 100 µm.**

**Figure S12. UMAP visualization of identified cell populations from the scRNA-seq dataset. Uniform Manifold Approximation and Projection (UMAP) plot of the integrated single-cell RNA sequencing (scRNA-seq) dataset from healing human skin wounds. Each point represents a single cell, clustered and color-coded based on its transcriptomic profile. Major cell populations were annotated based on canonical marker gene expression, including Fibroblasts, Endothelial cells, Keratinocytes, Lymphoid cells, Myeloid cells, and smaller clusters of Mast cells, Melanocytes, and Pericytes (Peri_SMC).**

**## Summary of Key Improvements**

**Figure S13. Sub-clustering analysis reveals heterogeneity within the wound fibroblast population. UMAP visualization of the computationally isolated fibroblast population from Figure S12. The fibroblasts were sub-clustered based on their transcriptomic profiles, revealing three distinct subpopulations: Remodeling Fibroblasts (FB_Remodeling), Adipogenic-like Fibroblasts (FB_AdipoLike), and Inflammatory Fibroblasts (FB_Inflammatory).**

**Figure S14. Dynamic changes in fibroblast subpopulation proportions during wound healing. Stacked bar chart showing the fractional abundance of the three fibroblast subpopulations (AdipoLike, Inflammatory, and Remodeling) in normal skin and at days 1, 7, and 30 post-injury.**

**Figure S15. Intercellular communication network in the healing wound at day 1. A network plot visualizing the cell-cell communication landscape inferred from the scRNA-seq dataset at day 1 post-injury. Each cell population is represented as a node, and the lines (edges) connecting them represent predicted signaling interactions. The thickness of each line is proportional to the strength or number of inferred interactions.**

**Figure S16. TGF-β1 enhances fibroblast migration in a scratch wound healing assay. Quantification of the scratch wound closure rate (%) for rat embryonic fibroblasts (REFs) after 24 hours of culture in medium with (TGF-β1⁺) or without (TGF-β1⁻) TGF-β1. Data points represent individual replicates, and bars represent the mean ± SD (n=3). Statistical significance was determined by a two-tailed unpaired Student’s t-test; ***p* < 0.01.**

**Figure S17. TGF-β1 enhances fibroblast migration in a Transwell assay. (Top) Representative microscopy images of crystal violet-stained rat embryonic fibroblasts (REFs) that migrated through the Transwell membrane. (Bottom) Quantification of the number of migrated cells per field after 24 hours of culture with (TGF-β1⁺) or without (TGF-β1⁻) TGF-β1. Data points represent individual replicates, and bars represent the mean ± SD (n=3). Statistical significance was determined by a two-tailed unpaired Student’s t-test; **p* < 0.05. Scale bars = 100 µm.**

**Figure S18. Schematic diagram and photographs (side and top views) of the custom-built apparatus used for burst pressure testing. To perform the test, the excised anterior abdominal wall is securely mounted on the device with the original incision site centered. Air pressure is then gradually increased from below until the tissue ruptures. The peak pressure at the point of failure is digitally recorded as the burst pressure.**

**Figure S19. (Left) Representative Western blot images and (Right) corresponding quantitative analysis of Collagen I (Col I), Collagen III (Col III), and α-smooth muscle actin (α-SMA) expression in tissue lysates from the Wound, Isotropic Control, and HARP-Perpendicular groups at day 14. Data are presented as mean ± SD (n=3). Statistical significance was determined by one-way ANOVA with Tukey's post hoc test. Significance levels are denoted as follows: *p < 0.05, **p < 0.01, and ***p < 0.001.**

**Figure S20. Representative images of Picrosirius Red stained tissue sections from the incision site of the Wound, Isotropic Control, HARP-Parallel and HARP-Perpendicular groups at day 14, viewed under polarized light. Scale bars = 200 µm.**

**Figure S21. Representative images of immunohistochemical (IHC) staining for α-smooth muscle actin (α-SMA) in tissue sections from the Wound, Isotropic Control,** **HARP-Parallel and HARP-Perpendicular groups at day 14. The HARP-Perpendicular group displays the most intense brown-positive staining, indicating robust myofibroblast-associated remodeling activity. In comparison, the Isotropic Control group shows moderate α-SMA expression, while the untreated Wound group shows the weakest staining. Scale bar = 200 µm.**

**Figure S22. Representative H&E-stained images of abdominal incision tissues at day 14. Images are shown for the Wound, Hydrogel, Hydrogel+Fibroblasts, Hydrogel+TGF-β1, and HARP groups. Hydrogel denotes the perpendicular hydrogel construct; Hydrogel+Fibroblasts and Hydrogel+TGF-β1 denote the corresponding constructs supplemented with fibroblasts or TGF-β1, respectively; HARP denotes the complete HARP-Perpendicular construct. Scale bar, 200 μm.**

**Figure S23. Representative Masson’s trichrome-stained images of abdominal incision tissues at day 14. Images are shown for the Wound, Hydrogel, Hydrogel+Fibroblasts, Hydrogel+TGF-β1, and HARP groups. Hydrogel denotes the perpendicular hydrogel construct; Hydrogel+Fibroblasts and Hydrogel+TGF-β1 denote the corresponding constructs supplemented with fibroblasts or TGF-β1, respectively; HARP denotes the complete HARP-Perpendicular construct. Scale bar, 200 μm.**

**Figure S24. Representative α-SMA immunohistochemical images of abdominal incision tissues at day 14. Images are shown for the Wound, Hydrogel, Hydrogel+Fibroblasts, Hydrogel+TGF-β1, and HARP groups. Hydrogel denotes the perpendicular hydrogel construct; Hydrogel+Fibroblasts and Hydrogel+TGF-β1 denote the corresponding constructs supplemented with fibroblasts or TGF-β1, respectively; HARP denotes the complete HARP-Perpendicular construct. Scale bar, 200 μm.**

**Figure S25. Representative polarized-light Picrosirius Red images of abdominal incision tissues at day 14. Images are shown for the Wound, Hydrogel, Hydrogel+Fibroblasts, Hydrogel+TGF-β1, and HARP groups. Hydrogel denotes the perpendicular hydrogel construct; Hydrogel+Fibroblasts and Hydrogel+TGF-β1 denote the corresponding constructs supplemented with fibroblasts or TGF-β1, respectively; HARP denotes the complete HARP-Perpendicular construct. Scale bar, 200 μm.**

**Figure S26. Sample-to-sample correlation analysis of bulk RNA-seq data. Pearson correlation heatmap of RNA-seq samples from the Wound and HARP-Perpendicular groups. The color scale indicates Pearson correlation coefficients between samples.**

**Figure S27. Heatmap of differentially expressed genes between Wound and HARP-Perpendicular tissues.Heatmap showing differentially expressed genes between the Wound and HARP-Perpendicular groups. Expression values are shown as row Z-scores.**

**Figure S28. GSEA of GO biological process gene sets.Dot plot showing GO biological process gene sets enriched in the Wound and HARP-Perpendicular groups. Positive normalized enrichment scores (NES) indicate enrichment in the HARP-Perpendicular group, whereas negative NES values indicate enrichment in the Wound group. Dot size represents gene set size, and color indicates NES.**

**Figure S29. GO biological process enrichment analysis of HARP-upregulated genes.Dot plot showing GO biological processes enriched among genes upregulated in the HARP-Perpendicular group. Dot size represents gene count, and color indicates the -log10 adjusted P value.**

**Figure S30. GO biological process enrichment analysis of HARP-downregulated genes.Dot plot showing GO biological processes enriched among genes downregulated in the HARP-Perpendicular group. Dot size represents gene count, and color indicates the -log10 adjusted P value.**

**Figure S31. KEGG pathway enrichment analysis of HARP-upregulated genes.Dot plot showing KEGG pathways enriched among genes upregulated in the HARP-Perpendicular group. Dot size represents gene count, and color indicates the -log10 adjusted P value.**

**Figure S32. KEGG pathway enrichment analysis of HARP-downregulated genes.Dot plot showing KEGG pathways enriched among genes downregulated in the HARP-Perpendicular group. Dot size represents gene count, and color indicates the -log10 adjusted P value.**

**Table S1. Parameters for the optimization of DAC synthesis.**

| No. | Temperature (°C) | Reaction time (h) | Molar ratio (MCC:NaIO₄) | pH |
| --- | --- | --- | --- | --- |
| 1 | 25 | 1 | 1:1 | 3 |
| 2 | 25 | 3 | 1:1.25 | 4 |
| 3 | 25 | 5 | 1:1.5 | 5 |
| 4 | 35 | 1 | 1:1.25 | 5 |
| 5 | 35 | 3 | 1:1.5 | 3 |
| 6 | 35 | 5 | 1:1 | 4 |
| 7 | 45 | 1 | 1:1.15 | 4 |
| 8 | 45 | 3 | 1:1 | 5 |
| 9 | 45 | 5 | 1:1.25 | 3 |

| Gene name | Direction | Sequence |
| --- | --- | --- |
| Col-I | Forward | CCCCCTCCCAGCCACAAAG |
|  | Reverse | TCTTGGTCGGTGGGTGACTCT |
| Col-III | Forward | CCAAACTCTATCTGA |
|  | Reverse | GGACTCATGAATAACA |
| α-SMA | Forward | AATGAGATGGCCACTGCCGC |
|  | Reverse | CAGAGTATTTGGGCTCCGGA |
| GAPDH | Forward | ACGACATCTTCGCTGCTGAG |
|  | Reverse | GGTCACCAGGCACGGTGTAC |

**Table S2. Primer sequences for qRT-PCR.**
